# No observable non-thermal effect of microwave radiation on the growth of microtubules

**DOI:** 10.1038/s41598-024-68852-3

**Published:** 2024-08-07

**Authors:** Greger Hammarin, Per Norder, Rajiv Harimoorthy, Guo Chen, Peter Berntsen, Per O. Widlund, Christer Stoij, Helena Rodilla, Jan Swenson, Gisela Brändén, Richard Neutze

**Affiliations:** 1 Department of Chemistry and Molecular Biology, University of Gothenburg, Gothenburg, Sweden; 2 Department of Physics, Chalmers University of Technology, Gothenburg, Sweden; 3 Monash Health Imaging, Monash Health, Clayton, VIC Australia; 4 Institution of Biomedicine, University of Gothenburg, Gothenburg, Sweden; 5 CSTechnologies, Växjö, Sweden; 6 Department of Microtechnology and Nanoscience, Chalmers University of Technology, Gothenburg, Sweden

**Keywords:** Perturbations, Cytoskeletal proteins

## Abstract

Despite widespread public interest in the health impact of exposure to microwave radiation, studies of the influence of microwave radiation on biological samples are often inconclusive or contradictory. Here we examine the influence of microwave radiation of frequencies 3.5 GHz, 20 GHz and 29 GHz on the growth of microtubules, which are biological nanotubes that perform diverse functions in eukaryotic cells. Since microtubules are highly polar and can extend several micrometres in length, they are predicted to be sensitive to non-ionizing radiation. Moreover, it has been speculated that tubulin dimers within microtubules might rapidly toggle between different conformations, potentially participating in computational or other cooperative processes. Our data show that exposure to microwave radiation yields a microtubule growth curve that is distorted relative to control studies utilizing a homogeneous temperature jump. However, this apparent effect of non-ionizing radiation is reproduced by control experiments using an infrared laser or hot air to heat the sample and thereby mimic the thermal history of samples exposed to microwaves. As such, no non-thermal effects of microwave radiation on microtubule growth can be assigned. Our results highlight the need for appropriate control experiments in biophysical studies that may impact on the sphere of public interest.

## Introduction

Microwaves span the electromagnetic spectrum from wavelengths of one millimetre to one meter (300 GHz to 300 MHz). Modern technology exploits this spectral domain with applications including mobile telephones, wireless LAN, Bluetooth, navigation radar, automobile radar, communications satellites, global positioning system (GPS), military radar targeting, microwave ovens, radio astronomy and medical applications. Whether or not there may be negative health effects arising from being constantly immersed in radiation within this frequency domain has been the subject of considerable debate and controversy^1,2^. Public interest is particularly high concerning the widespread use of mobile telephones and telephone transmitters^3,4^. One well known effect of microwaves is that they induce heating. For example, the guidelines for microwave devices are focused on the thermal response and baselined at levels far below what is known to be harmful^5^. Conversely, because thermal effects are strong, any non-thermal effects of microwave (non-ionizing) radiation may be overlooked.

Theoretical suggestions for protein resonances in this frequency domain^6–8^ have long been proposed but lack conclusive supporting experimental evidence. The existence of such resonances has also been challenged on the basis that vibrational damping will suppress such processes^9^. Protein normal modes may be selectively enhanced in lysozyme crystals exposed to radiation in the THz domain^10^, but an earlier study of the influence of microwave radiation on lysozyme crystals did not reveal non-thermal structural effects^11^ and small angle X-ray scattering studies of the effect of THz radiation on proteins did not reveal structural changes^12^. Other claims of non-thermal effects of microwave radiation observed on cells or macromolecules often rely heavily on knowledge of microwave physics for their interpretation^7,13–15^ and consequently struggle to convince across interdisciplinary barriers. Other studies on living cells do not show any non-thermal effects^16^, and one high-profile article that claimed a non-thermal heat-shock response to microwave radiation^17^ was later retracted because the experimental conditions actually involved a modest temperature rise^18^. For these reasons, the studies of non-thermal effects of microwaves on cells have not been reproducible and explanations have been offered to describe this problem^19^.

Here we examine the influence of an applied microwave field on the growth of microtubules by monitoring time-dependent changes in the sample’s turbidity^20^. Tubulin is a protein that is integral to the eukaryotic cytoskeleton and exists in solution as a dimer of two globular proteins, α and β tubulin. These dimers assemble into dynamical polymeric tubes in the presence of Guanosine triphosphate (GTP, a major cellular metabolite) that are approximately 24 nm in diameter and can extend up to several micrometres in length. Microtubules are critical for cellular organization, motility, transport and mitosis^21^. Microtubules are also dynamically instable due to a constant process of assembly and disassembly, which leads to periods of rapid growth interrupted by periods of rapid shortening. The growth of microtubules from tubulin dimers can be described as comprising three phases: nucleation, elongation and saturation^22^. During nucleation, new microtubule aggregates are generated from tubulin dimers. As these nuclei grow, they pass through their so-called least-stable complex, after which they enter the elongation phase by the addition of dimers onto these previously formed nuclei. At saturation the rate of growth slows as the number of free tubulin dimers become relatively scarce. An important mechanistic idea underpinning this process is that microtubules are believed to assemble in vitro via the formation of small sheets which grow wider and longer and eventually fold into tubes once they achieve their full complement of approximately thirteen proto-filaments (thirteen tubulin dimers per turn) in width^22^. Since microtubules are highly polar, and in some respects can be imagined as having characteristics similar to radio antennae but on a micrometre scale, they have been suggested to be sensitive to non-thermal effects of microwave radiation^23,24^.

To investigate non-thermal influences of microwave radiation on the growth of microtubules, we measured the polymerization of tubulin in response to electromagnetic radiation 3.5 GHz, 20 GHz and 29 GHz in frequency. These electromagnetic fields were applied using a waveguide with a plate separation of 1 mm, and the peak-to-peak voltages ranged from -2 V to 2 V (specific details and Specific Absorption Rate (SAR) values given in Table 1). These three frequencies also span the domain used by 5G networks (0.6–29 GHz). Changes in sample optical density were followed as a time-dependent increase in turbidity used as a proxy for microtubule formation^25^. An infrared camera characterized the spatial distribution of sample heating, with the sample’s optical density (O.D.) being measured at the point where heating from the microwave field was strongest. Simultaneous measurements of the sample’s optical density and temperature allowed both thermal and non-thermal responses to be characterized. We observe that the growth curves for microtubules in samples exposed to 20 GHz and 29 GHz, radiation depart from the growth trajectories observed at the same temperature when there is no exposure to microwaves, whereas samples exposed to 3.5 GHz radiation do not show this discrepancy. These apparent non-thermal effects, however, could be reconciled with additional control studies in which the thermal history of the sample as it entered the microwave field was mimicked. These findings illustrate how false conclusions may arise if subtleties within the experimental design are not appreciated and emphasises the need for careful design of control experiments given the widespread concerns regarding the effect of microwaves on public health.

**Table 1:** Waveguide characterization and electromagnetic field exposure.

Waveguide characterization		
	S21 (transmitted) [dB]	S11 (reflected) [dB]
Measured 3.5 GHz buffer	−1.49	−8.45
Measured 3.5 GHz empty	−1.31	−8.37
Measured 20 GHz buffer	−10.73	−11.88
Measured 20 GHz empty	−3.40	−12.52
Measured 29 GHz buffer	−16.48	−9.30
Measured 29 GHz empty	−6.49	−4.55
Simulated 3.5 GHz buffer	−0.08	−22.92
Simulated 3.5 GHz empty	−0.04	−28.29
Simulated 20 GHz buffer	−7.16	−10.56
Simulated 20 GHz empty	−1.43	−17.51
Simulated 29 GHz buffer	−11.67	−13.37
Simulated 29 GHz empty	−2.79	−7.05

Electromagnetic field and Specific absorption rate in sample		
	V_peak_/m*	SAR [W/kg]
3.5 GHz 166 mW input	600	300
20 GHz 26mW input	1000	800
20 GHz 66 m W input	1300	1400
20 GHz 166 mW input	1800	2700
29 GHz 166 mW input	1900	2900

*Peak electromagnetic field strengths at the point of optical density measurements are calculated after subtracting the power losses due to reflection (S11) and transmission (S21) through the output port of the waveguide.

## Results

### Exposure of samples to microwave fields

**Figure 1:**
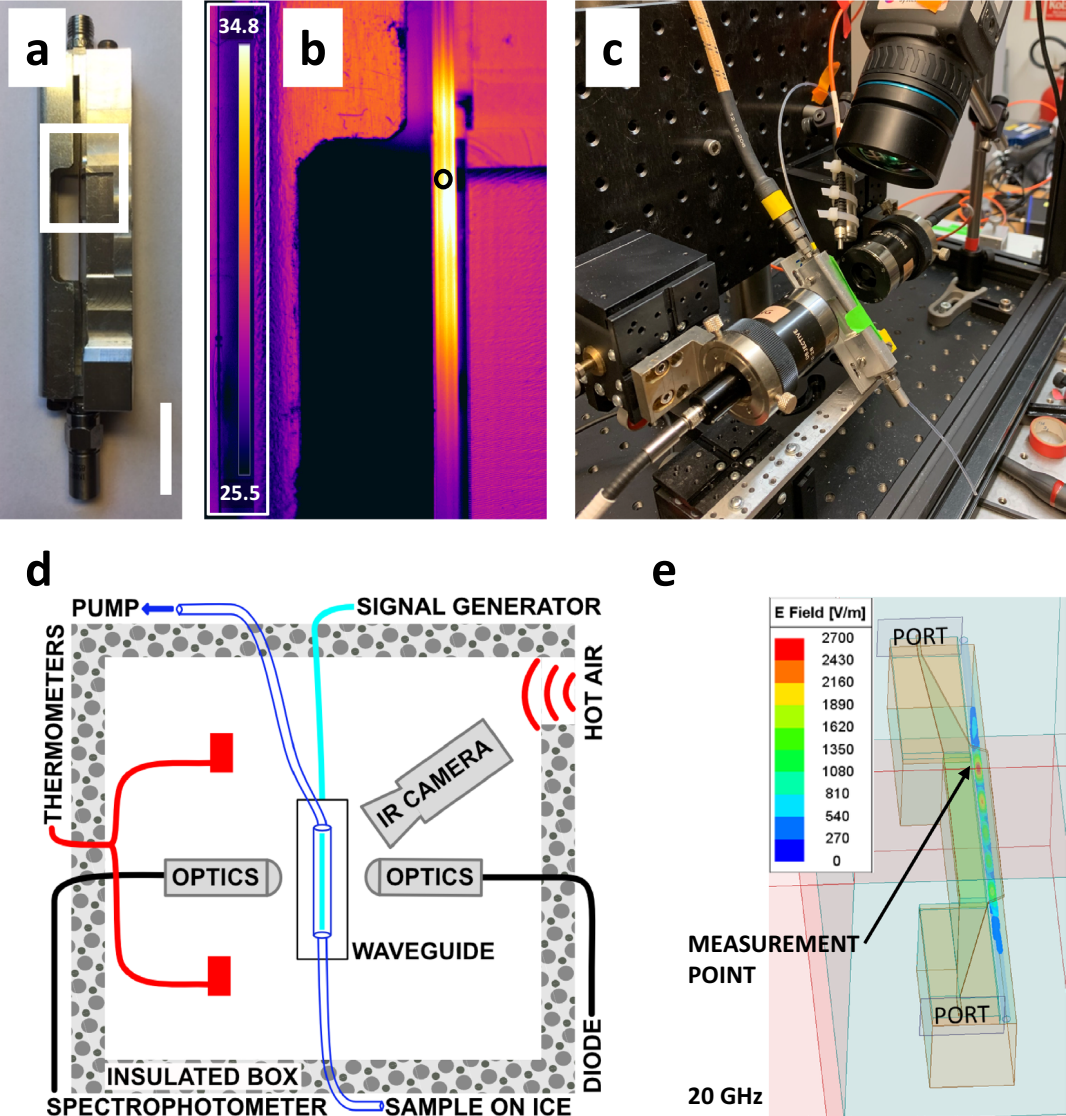
Experimental apparatus used to measure changes in optical density (O.D.) of microtubule samples during exposure to 3.5–29 GHz AC fields. (**a**) Photograph of the waveguide used to deliver 3.5 to 29 GHz radiation onto a 1 mm diameter quartz capillary in which the sample was held. The white box indicates the region shown in the next panel. The white bar represents 2 cm. (**b**) Infrared image of the sample within a quartz capillary completely filled with water during exposure to 20 GHz radiation. For this image the IR camera was mounted directly in front of the flow cell. The measurement spot during measurements is marked with a black circle. Colour bar inset shows temperature profile increase from room temperature. (**c**) Photograph of the experimental setup used to record the O.D. at 365 nm with time during simultaneous exposure to microwaves. This entire setup was enclosed within a temperature-controlled box. An infrared camera (FLIR thermographic) was used to monitor the temperature of the sample at the position at which its O.D. was recorded. (**d**) Schematic of the thermally insulated experimental setup which shows microspectrophotometer optics and connections to diode and spectrophotometer, positioning of the IR camera, hot air inlet, thermometers to measure ambient temperature, tubing connecting sample reservoir with the suction pump and electromagnetic signal generator. (**e**) Simulation of electric field inside the capillary for a 1 W input with a frequency of 20 GHz. The sample capillary is shaded light blue. The point of optical density measurement and connector ports are marked. The electric field varies along the sample in the capillary and the point at which the optical density was measured was located where the field strength was strongest. Field strength scales linearly with applied voltage.

We aimed to characterise the response of microtubules to an applied oscillating electromagnetic field. Having a background in X-ray scattering^26,27^, we designed a device which would support both light-scattering and X-ray scattering measurements. To this end a parallel-plate waveguide-based flow-cell was purpose built to deliver AC fields onto a quartz capillary containing the sample (Fig. 1). Both waveguide plates were 64 mm long, 5 mm wide and were separated by just over 1 mm. The waveguide was shaped so that only the central part of the waveguide, 25 mm long, ran adjacent to the capillary. A coaxial cable fed the GHz fields from a signal generator through the waveguide, which was terminated on a 50 Ω resistor. The waveguide was designed to work optimally within the frequency domain correlating with peak dielectric losses in water, which at room temperature is close to 20 GHz^28^.

An unavoidable consequence of sampling the effects of electromagnetic fields applied in the microwave domain is that samples are heated. Conversely, the observation of sample heating provides confidence that the device efficiently delivers microwaves onto the sample. To quantify this effect, we used a FLIR thermographic infrared camera to map the extent of water heating within the quartz capillary (Fig. 1b**, **Supplementary Figure S1). This allowed us to establish that the energy transfer from the AC field generator to the sample was maximal near 20 GHz, where both the microwave generator and the coupling were efficient. Moreover, by imaging the microwave heating profile using the thermographic infrared camera, we could record the sample’s optical density at the point where the heating was maximal. Changes in light-scattering in the microwave exposed quartz capillary were then measured using a microspectrophotometer that was similar to an earlier design^29^ (Fig. 1c, d). The majority of experiments presented here used 20 GHz radiation, but data were also recorded at 3.5 GHz and 29 GHz. Full wave electromagnetic simulations of the device for these three different frequencies show a standing wave pattern in the electromagnetic field distribution in the sample (Fig. 1e, Supplementary Fig. S2a, b). Weak oscillatory features are also visible to some extent by thermal imaging for the two higher frequency sets, although the pattern is more diffuse due to thermal diffusion (Supplementary Fig. S1a, d–e). Simulated S21 and S11 parameters show a similar frequency dependence as the measured values (Supplementary Fig. S2c, d), although the simulations slightly underestimate the losses.

20 GHz measurements were conducted with three different power output levels from the signal generator, 15 dBm (32 mW), 19 dBm (80 mW) and 23 dBm (200 mW). A fraction of the signal is lost in the coaxial cable from the signal generator to the waveguide, and this was measured as 0.8 dBm. As such, the input into the waveguide is lowered from the above values to become 26 mW, 66 mW and 166 mW respectively. By assuming that losses between the connector of the cable and the transmission line into the waveguide are negligible, assuming that the electric field strength varies linearly along the length of the waveguide, and by measuring the transmitted (S21) and reflected (S11) scattering parameters of the waveguide (Supplementary Fig. S2c, d) with both an empty capillary and when the capillary is filled with sample buffer, we could estimate the electromagnetic field at the sample position. The power estimated at the point at which the optical density was measured and its peak voltage are listed in Table 1 as a function of the measured input power. For an input frequency of 20 GHz and 166 mW input power, the electromagnetic field at the point of measurement is approximately 1.8 kV/m, which compares with the nominal input voltage of 4.1 kV/m.

The SAR within the sample can be calculated using the formula SAR = σ ∙E^2^/ρ. The buffer conductivity (σ) is measured to be 0.8 S/m and the sample density (ρ) is assumed to be that of water. It has been reported that polymerized microtubules can increase the conductivity of a solution by several percent, but we do not include this in our SAR estimate^30^. The calculated SAR values range between 300 and 2900 W/kg (Table 1). Depending upon which guidelines one compares to, which are different for different parts of the body and different frequencies, these calculated values vary from one to several orders of magnitude above the guidelines for human exposure^5^. For example, the whole-body average over time is given as a maximal exposure of 0.08 W/kg but local occupational exposures for brief intervals may allow exposures of up to 100 W/kg^5^.

### Data collection and analysis

GTP is required for tubulin dimers to polymerize, but they will not polymerize until the temperature is raised above a critical temperature^31^, which is approximately 18–19 °C at the sample concentrations used for this study. This effect is used in functional assays since tubulin samples can be mixed with GTP while on ice, which arrests their nucleation and growth. As aliquots of these samples are pumped through the flow-cell to the measurement position, they are warmed by the ambient temperature of the surrounding environment and this temperature jump initiated the polymerization reaction. After reaching the point of measurement, each sample aliquot (5 μl) was held stationary during the experiment, and was then replaced with a fresh 5 µl sample for a fresh measurement. Solubilized samples of tubulin dimers are almost completely transparent to 365 nm light, whereas samples of microtubules are opaque. This allows tubulin polymerization to be followed as the relative turbidity of the sample increases with time^25,31^. We followed this dynamical process by recording the sample’s optical density (O.D.) with time through a 1 mm capillary using a microspectrophotometer^29^ (Fig. 1c, d).

In our waveguide, the application of 20 GHz microwave radiation onto the sampled position with an input power of 66 mW and 166 mW resulted in the sample being heated by approximately 4 °C and 7 °C respectively. It was therefore necessary to enclose the apparatus within a box (Fig. 1d) and to set the ambient air temperature within this box lower than the target sample temperature at the position of observation by a compensatory amount (ie., 4 °C lower for 66 mW and 7 °C lower for 166 mW input power). The 29 GHz input heated samples to a very similar extent to 20 GHz input, whereas neither the 20 GHz with input power of 26 mW nor the 3.5 GHz microwave radiation visibly heated the sample (Supplementary Figure S1a). Throughout all light-scattering measurements, the temperature at the sample position was measured using the thermographic infrared camera and the temperature values reported for all data points correspond to the temperature at the sampled position, and consequently include heating from both the surrounding environment and the microwave radiation (Table 2, *T*_*f*_*—T*_*i*_). Because the growth of microtubules is a stochastic process, with their nucleation being relatively slow before a more rapid elongation phase, there may be considerable run-to-run variability in the growth curves^22,32^. We therefore repeated each data-point on average 22 times (Table 2, $N$), although there was variation in the sample of specific runs due to outlier rejection. Data were initially rejected when we had incomplete O.D. trajectories due to bubbles in the light path. Subsequent outlier rejections were done with the MAD method, a method similar to Z-score but based on medians rather than means^33^.

**Table 2:** Average and standard error in measured parameters from experimental data.

	$N$	*T*_*f*_-*T*_*i*_ (°C)	$O.D.$	$\frac{{\sigma }_{\text{O}.\text{D}.}}{\sqrt{N-1}}$	${t}_{10}$(s)	$\frac{{\sigma }_{{t}_{10}}}{\sqrt{N-1}}$	$b$	$\frac{{\sigma }_{b}}{\sqrt{N-1}}$
31.9 °C	20	0.62	1.06	0.03	57.3	4.0	2.04	0.06
34.9 °C	22	0.84	1.36	0.04	25.8	1.8	2.21	0.05
39.1 °C	18	0.97	1.58	0.03	10.3	0.5	2.06	0.06
3.5 GHz 166 mW	15	0.86	1.29	0.03	18.9	0.6	2.11	0.06
20 GHz 26 mW	20	1.16	1.37	0.03	18.1	0.7	2.15	0.05
20 GHz 66 mW	24	2.31	1.37	0.04	21.6	1.3	2.42	0.04
20 GHz 166 mW	26	4.64	1.49	0.03	20.7	0.5	2.98	0.04
29 GHz 166 mW	27	4.72	1.37	0.03	23.7	0.9	3.06	0.05
IR heating	30	3.02	1.93	0.01	11.8	0.3	2.86	0.04
Airflow heating	17	8.31	1.69	0.03	17.2	0.9	2.91	0.11

*N* is the number of repeats after outlier rejection.

*T*_*i*_ is the initial temperature and *T*_*f*_ is the final temperature in the IR camera measurement.

*O.D.* is the final optical density of the sample.

*t*_*10*_ is the time it takes to reach 10% of the final *O.D*

*b* is the exponent of the natural logarithm of the growth phase.

**Figure 2:**
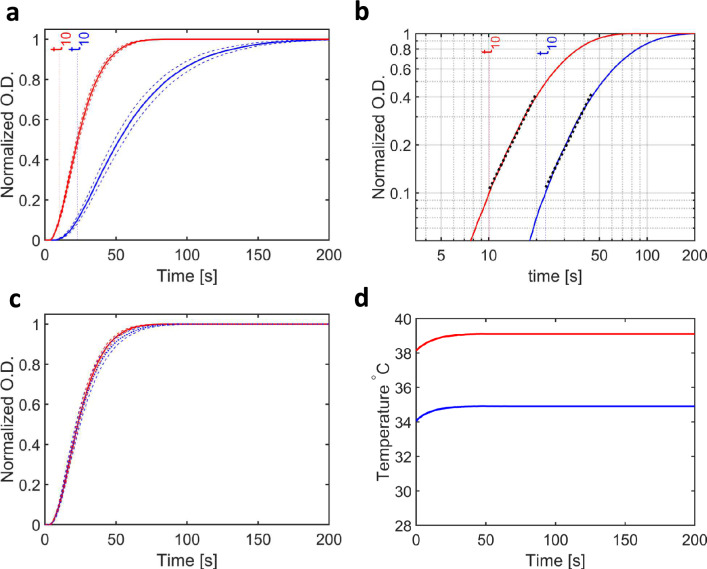
Measured changes in O.D. from samples of tubulin during the growth of microtubules. (**a**) Turbidity measurements ($O.D$.) with time ($t$) for samples exposed to a constant ambient temperature of 34.9 °C (blue line) and 39.1 °C (red line). Error bars represent the standard error, $\sigma /\sqrt{N-1}$, where $\sigma$ is the standard deviation of a set of $N$ measurements. These data have been normalized to have their end-point $O.D. =1$. (**b**) Plot of $O.D.$ versus time on a log–log scale. These curves were approximately linear between O.D. = 0.1 and O.D. = 0.4 and the slope of this line (black dots) yielded the $b$-value (Eq. 2). Since both lines are approximately parallel, a Mann–Whitney U-test comparing $b$-values from each set of runs showed no significant difference (Table 3). (**c**) Plot of the two curves shown in A after stretching in time such that the slower curve (blue line) superimposes on the faster curve (red line) with matching ${t}_{50}$ values. (**d**) Plot of the temperature measured at the sample position using the thermographic camera. In the case of homogeneous (ambient) heating, the temperature difference between the two measurements is almost constant throughout these experiments.

Representative measurements of the time dependence of the change in the sample’s $O.D.$ are shown in Fig. 2a, where data were measured using 10% (by volume) glycerol, 10 mg/ml tubulin (91 μM), and 2 mM GTP, with the temperature at the sample position 34.9 ± 0.3 °C and 39.1 ± 0.4 °C. These data have been normalized to have an initial optical density ($O.D.$) of zero and a final $O.D.$ of unity. Error bars in these curves are given as the standard error of the mean (*ie.* $\sigma /\sqrt{N-1})$, where $\sigma$ is the standard deviation and $N$ is the number of repeats of each of the measurements. These data follow the approximate sigmoidal curves typically associated with microtubule nucleation, growth and saturation^22,25,31^.

In the initial phase it is possible to approximate microtubule growth using a power law^25^

1 $$O.D.\left( t \right) = A \cdot \left( {\frac{t}{{t_{u} }}} \right)^{b}$$

where $A$ is a constant, $b$ is the power law exponent, and ${t}_{u}$ is a unit of time introduced in order to keep the equation dimensionless. Taking the natural logarithm of both sides gives:

2 $$\ln \left( {O.D.\left( t \right)} \right) = \ln \left( A \right) + b \cdot \ln \left( t \right) - b \cdot {\text{ln}}\left( {t_{u} } \right)$$

and a plot of $ln(O.D)$ vs *ln(t)*  should yield a straight line with slope $b$. Moreover, the point at which the $O.D.$ reaches 10% of its maximum value, ${t}_{10}$, may be read from the experimental curve directly. The same data presented in Fig. 2a are redrawn in Fig. 2b using logarithmic axes and yield approximately straight lines over the domains $0.1 \le O.D. \le 0.4$, where this linear fit is represented using dotted lines.

### Comparison of microtubule growth curves at different temperatures

The overriding goal of this work is to establish if measurements of the growth of microtubules show statistically significant differences in their behaviour when exposed to microwave radiation. In drawing statistical comparisons between experimentally measured curves, we examine three parameters: the final $O.D.$, the ${t}_{10}$ values, and the $b$ values extracted from fitting Eq. 2 to the experimental data. From these parameters extracted from $N$ repeats for each condition (Table 2), the results from two experimental conditions were compared using the Mann–Whitney U-test (also called the Wilcoxon rank sum test), which evaluates the hypothesis that two sets of measurements have different medians. The Mann–Whitney U-test is a non-parametric test and therefore does not require that data are normally distributed. This statistic test was chosen since we could not conclude that all parameters for all experimental conditions were normally distributed (Supplementary Figure S3 illustrates the results of normality checks using Anderson–Darling, One-sample Kolmogorov–Smirnov, Lilliefors and Jarque–Bera tests by labelling dataset histograms red if they fail at least one of these tests). Because microtubule nucleation and growth are stochastic processes, because we used a small volume of sample (~ 5 μl) in our flow-cell in order to optimize the microwave field-strength, and because the kinetics of microtubule growth are sensitive to multiple parameters^25^ which may fluctuate slightly from run to run, there was considerable variation in these parameters from one measurement to the next. We therefore take a $p$-value < ${10}^{-3}$ as indicating a statistically significant difference, and this condition is usually regarded as stringent. As is illustrated in Table 3, a weaker requirement of $p$-values < $0.05$ would not change any of our major conclusions in any substantial way.

**Table 3:** Mann–Whitney U test comparison of growth curve data.

		Measured			*Stretched
		$O.D.$	${t}_{10}$	$b$	${t}_{10}$
Comparison of samples heated (homogeneously) to different temperatures					
31.9 °C *vs* 34.9 °C	$p$-values	2 × 10^–5^	2 × 10^–7^	0.02	0.16
31.9 °C *vs* 39.1 °C		5 × 10^–8^	2 × 10^–7^	0.70	0.23
34.9 °C *vs* 39.1 °C		6 × 10^–4^	8 × 10^–8^	0.10	0.35
Microwave exposed measurements versus homogeneous (ambient) heating					
34.9 °C *vs* 3.5 GHz 166 mW	$p$-values	0.09	0.03	0.29	0.65
34.9 °C *vs* 20 GHz 25 mW		0.99	0.002	0.52	0.66
34.9 °C *vs* 20 GHz 66 mW		0.41	0.03	6 × 10^–4^	0.51
34.9 °C *vs* 20 GHz 166 mW		0.02	0.07	5 × 10^–9^	0.05
34.9 °C *vs* 29 GHz 166 mW		0.92	0.52	4 × 10^–9^	0.03
Microwave exposed measurements versus heating from an air stream					
Air heating *vs* 3.5 GHz 166 mW	$p$-values	1 × 10^–6^	0.11	6 × 10^–5^	0.002
Air heating *vs* 20 GHz 26 mW		2 × 10^–6^	0.74	3 × 10^–5^	0.001
Air heating *vs* 20 GHz 66 mW		6 × 10^–5^	0.17	5 × 10^–4^	0.13
Air heating *vs* 20 GHz 166 mW		5 × 10^–4^	0,001	0.89	0.84
Air heating *vs* 29 GHz 166 mW		5 × 10^–7^	4 × 10^–4^	0.16	0.89
Air heating *vs* 34.9 °C		4 × 10^–6^	0,002	3 × 10^–5^	0.08
Microwave exposed measurements versus infrared heating					
IR heating *vs* 3.5 GHz 166 mW	$p$-values	8 × 10^–9^	2 × 10^–8^	4 × 10^–8^	2 × 10^–4^
IR heating *vs* 20 GHz 26 mW		1 × 10^–9^	5 × 10^–9^	3 × 10^–9^	4 × 10^–4^
IR heating *vs* 20 GHz 66 mW		7 × 10^–11^	5 × 10^–8^	2 × 10^–8^	0.45
IR heating *vs* 20 GHz 166 mW		5 × 10^–11^	8 × 10^–11^	0.09	0.013
IR heating *vs* 29 GHz 166 mW		1 × 10^–11^	4 × 10^–11^	0.003	0.15
IR heating *vs* 34.9 °C		4 × 10^–10^	7 × 10^–10^	2 × 10^–9^	0.20

*Time-parameter scaled so that the mean ${t}_{50}$ value was numerically the same as for the control.

**Figure 3:**
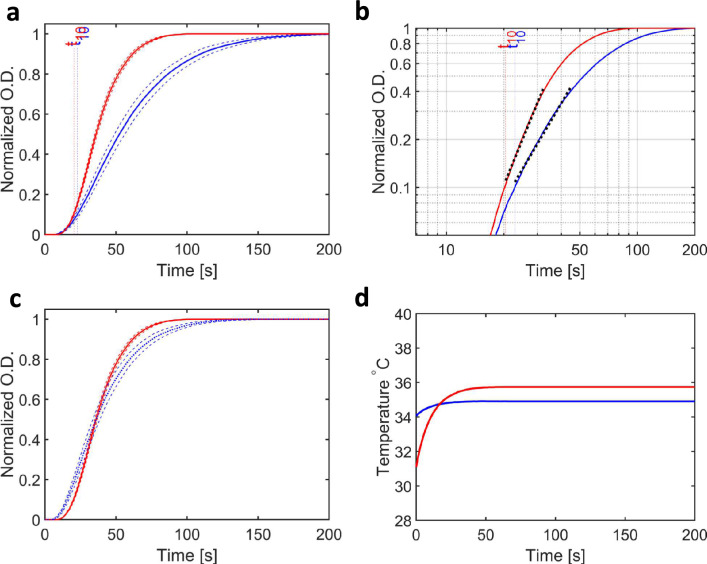
Measured changes in O.D. from samples of tubulin exposed to microwaves during the growth of microtubules. (**a**) Turbidity (normalized $O.D.$) with time ($t$) for samples exposed to a constant ambient temperature of 34.9 °C (blue line) and when exposed to 20 GHz microwave radiation (red line). (**b**) Plot of $O.D.$ versus time on a log–log scale. Since these lines are not parallel, a Mann–Whitney U-test showed a significant difference when comparing $b$-values from the two sets of data (Table 2). (**c**) Plot of the two curves shown in A after stretching in time such that the faster curve (red line) superimposes on the slower curve (blue line) with matching ${t}_{50}$ values. (**d**) Plot of the temperature measured at the sample position using the thermographic camera. A larger change in temperature with time was associated with the microwave measurements.

To illustrate this procedure, consider the comparison between the behaviour of microtubule growth curves at the two temperatures illustrated in Fig. 2. As seen in Table 2, the final $O.D.$ and ${t}_{10}$ values vary as the temperature is varied, yet the measured $b$-values were relatively consistent within the experimental uncertainty of the mean values of these parameters. These observations are reflected in conclusions drawn from the Mann–Whitney U-test (Table 3), which show that the experimental traces from the separate measurements yield $p\le {10}^{-3}$ for the $O.D.$ and the ${t}_{10}$ values when comparing these measurements between any two of the three temperatures: 31.9 ± 0.6 °C, 34.9 ± 0.3 °C and 39.1 ± 0.4 °C. For example, the mean final $O.D.$ at 39.1 °C is 49% higher than that resulting at 31.9 °C, and 16% higher than for measurements performed at 34.9 °C. This statistically significant increase in $O.D.$ with temperature is consistent with earlier observations^25^. Similarly, since all reactions are accelerated as the temperature increases, the ${t}_{10}$ values will be considerably lower at elevated temperature, since the kinetics of the reaction are faster^25^. This is measured experimentally with ${t}_{10}$ = 57.3 ± 4.0 s at 31.9 °C, ${t}_{10}$ = 25.8 ± 1.8 s at 34.9 °C and ${t}_{10}$ = 10.3 ± 0.5 s at 39.1 °C (Table 2). Despite these differences, comparison of the $b$-values extracted using Eq. 2 for data recorded at 34.9 °C and 39.1 °C yielded a $p$-value of 0.10, which is not considered significantly different. This is illustrated in Fig. 2b since the apparent slopes of the two linear-fits to the experimental data on log($O.D.$) *vs.* log($t$) plots are almost parallel. Indeed, Fig. 3 of reference^25^ shows that log($O.D.$) *vs.* log($t$) plots yield parallel lines under a great variety of experimental conditions. The statistical test yielded a $p$-value of 0.02 when comparing b-values at 31.9 °C and 34.9 °C, which fails out threshold of $p\le {10}^{-3}$, but potentially indicate that the ratios of the various forwards and backwards rate-constants describing tubulin polymerization^31,34^ may diverge slightly at the lower temperature.

Although microtubule polymerization is a multi-step process, including a nucleation phase, an elongation phase and a saturation phase, if the kinetics of all participating forward and backward reactions co-vary with temperature, then the shape of the resulting sigmoidal curve will be independent of temperature. One test for this hypothesis is to scale the time-variable for one set of measurements, such that the value when the average of two separate measurements reaches their mid-point $O.D.$ (called ${t}_{50}$) is numerically the same for both measurements (Fig. 2c). We can then perform a Mann–Whitney U-test on the resulting distribution of ${t}_{10}$ variables after time has been “stretched” in order to make this comparison. This procedure changes neither the $b$-value nor the $O.D.$. The statistical test results after this manipulation are given in Table 3 under the column heading “Stretched”. For example, when time-scaling was imposed and data recorded at 31.9 ± 0.6 °C, 34.9 ± 0.3 °C and 39.1 ± 0.4 °C were compared, the $p$-values for ${t}_{10}$ increased from < 10^–6^ to 0.16, 0.23 and 0.35, none of which indicate a statistically significant difference. Thus this “time-stretching” procedure demonstrates that the ratio of ${t}_{50}$ to ${t}_{10}$ is statistically independent of the temperature at which these measurements were performed.

### Comparison of microtubule growth curves when exposed to microwaves

With these tools of analysis illustrated above, we are in a position to compare the growth curves of microtubules when exposed to microwave radiation with control studies of samples held at constant temperature. Figure 3 illustrates how the presence of microwave fields change the growth-curves when using 20 GHz radiation applied at 166 mW (1.8 kV/m, SAR 2.7 kW/kg). Although the growth curve is qualitatively similar (Fig. 3a), a log($O.D.$) vs log($t$) plot reveals that the slopes of these graphs are not parallel (Fig. 3b). This is quantified in Table 2, in which the average $b$-value at 34.9 °C of 2.21 ± 0.05 increases to 2.98 ± 0.04 as the microwave field is applied. Moreover, the Mann–Whitney U-test of the $b$-values for these runs give $p$ < 10^–8^, which is a statistically significant difference. This difference is visualized more easily by applying time-stretching in order to match ${t}_{50}$ for the two measurements (Fig. 3c), which yields a growth curve when 20 GHz radiation is applied that does not superimpose well with the 34.9 °C control which is not exposed to microwave radiation. In this case, however, the Mann–Whitney U-test after stretching yields $p$ = 0.05 for the comparisons on ${t}_{10}$, which we do not consider to be sufficiently low to claim a statistically significantly difference given the uncertainties in the mean after stretching.

Very similar results are recovered for measurements at 29 GHz using 166 mW of power (1.9 kV/m, SAR 2.9 kW/kg), for which again the average $b$-value increased to 3.06 ± 0.05 when a microwave field was applied. As previously, the statistical test yields $p$ < 10^–8^ when comparing $b$-values, but $p$ = 0.03 for ${t}_{10}$ after time-stretching. As such, for both 20 GHz and 29 GHz radiation using a power of 166 mW there is a statistically significant change in the measured $b$-values. Moreover, for the lower applied powers of 26 mW (1 kV/m, SAR 0.8 kW/m) and 66 mW (1.3 kV/m, SAR 1.4 kW/kg) there is a shift in the mean b-values from 2.21 ± 0.04 for the constant temperature measurements at 34.9 °C, to 2.15 ± 0.05 when 26 mW of 20 GHz radiation is applied, and to 2.42 ± 0.04 when exposed to 66 mW of 20 GHz radiation. In this case the shift in $b$-values is significant for the 66 mW field according to the criteria used here ($p$ = 0.0006 according to the statistical test, Table 3) and there is a consistent trend to lower $p$-values with increasing input power. The exposure of electromagnetic fields with a frequency of 3.5 GHz (600 V/m, SAR 0.3 kW /kg) doesn not alter the b-value in a statistically significant way. We therefore conclude that the application of microwave radiation of 20 GHz or 29 GHz in frequency with an input power of 166 mW onto a volume of approximately 5 μl leads to a measurable change in the power-law describing the growth of microtubules. This is striking since the measurement of the growth of microtubules repeatedly showed that the power-law was conserved over a large variety of experimental conditions^31^.

### Comparison of microtubule growth curves exposed to microwaves, infrared laser and airflow heating

In the previous section, we recovered statistically significant observable differences between the measured growth curves for microtubules raised to constant temperature relative to those exposed to microwaves. These observations raise the question whether or not this effect is the result of a previously unobserved non-thermal effect of microwave radiation that perturbs the kinetics of nucleation or growth, or if there may be more subtle thermal effects that warrant further investigation. As noted above, one consideration is that, because of the heating effect of the microwaves, the temperature of ambient environment surrounding the sample must be set a few degrees lower than in the control studies. This means that the thermal history of the sample is slightly different between the experiment using microwaves and the control, and this can be seen by comparing their temperature traces (Figs. 2d, 3d). Specifically, the sample’s $O.D.$ is measured from the moment the sample reaches the position where the light from the microspectrophotometer is incident upon the capillary. Since the flow-rate from the sample on ice to the measurement position is the same for all measurements, there is a period of approximately seven seconds (transfer line of 73 μl of 1 mm inner diameter pumped at 10 μl/s) during which the sample which was held at 0 °C increases to the ambient temperature. Because the ambient temperature is lower for the microwave exposed studies, the sample is slightly cooler when it arrives at the position of measurement when exposed to microwave radiation than for the control studies, and this is apparent from the measured temperature traces at the sample position (Fig. 3d).

**Figure 4:**
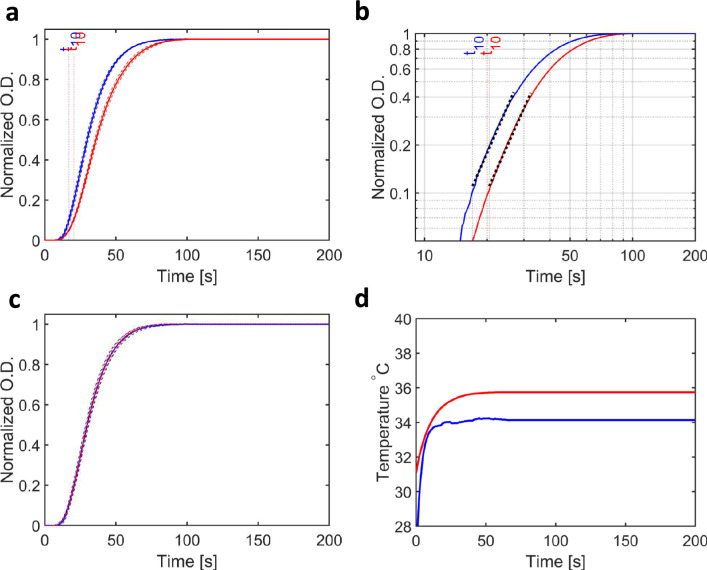
Measured changes in $O.D.$ for samples of tubulin exposed to microwaves compared to samples heated by a laminar of warm air during the growth of microtubules. (**a**) Turbidity (normalized $O.D.$) with time ($t$) for samples exposed to 20 GHz microwave radiation (red line) and when exposed a local thermal gradient (blue line). (**b**) Plot of $O.D.$ versus time on a log–log scale. Since both lines are approximately parallel, a Mann–Whitney U-test comparing $b$-values from the separate runs showed no significant difference (Table 2). (**c**) Plot of the two curves shown in A after stretching in time such that the air heated curve (blue line) superimposes on the microwaved exposed curve (red line) with matching ${t}_{50}$ values. (**d**) Plot of the temperature measured at the sample position using the thermographic camera. A comparable change in temperature with time was associated with the two exposure protocols.

**Figure 5:**
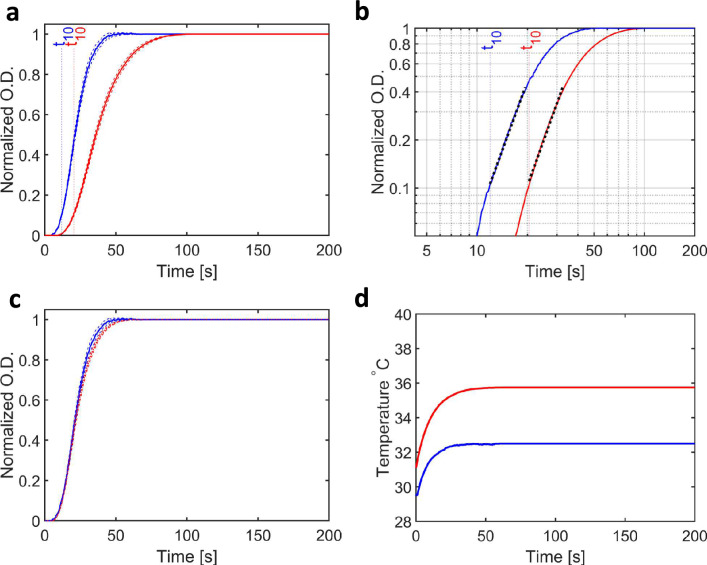
Measured changes in O.D. from samples of tubulin exposed to microwaves compared to samples heated by an IR laser during the growth of microtubules. (**a**) Turbidity (normalized $O.D.$) with time ($t$) for samples exposed to a 20 GHz microwave radiation (blue line) and when exposed a local thermal gradient (red line). (**b**) Plot of $O.D.$ versus time on a log–log scale. Since both lines are approximately parallel, a Mann–Whitney U-test comparing $b$-values from the separate runs showed no significant difference (Table 2). (**c**) Plot of the two curves shown in A after stretching in time such that the IR laser heated curve (blue line) superimposes on the microwave exposed curve (red line) with matching  ${t}_{50}$ values. (**d**) Plot of the temperature measured at the sample position using the thermographic camera. Since the IR images were recorded on the opposite side of the capillary as the IR laser used for heating, it is possible that the induced heating is not homogeneous across the capillary and its value is therefore underestimated. Both experimental protocols show a similar change in temperature with time.

To account for this discrepancy in the thermal history of the sample, we designed a control study in which the sample was raised to the end point temperature step-wise: first by holding the flow-cell in an environment in which the ambient temperature was above zero but lower than the target measurement temperature, and then using either a focused air-flow to heat the sample at the measurement position (Fig. 4), or by using an infrared laser to heat the sample in a focused spot and thereby raise the sample’s temperature to the target value (Fig. 5). As seen in Figs. 4d and 5d, this strategy meant that the temperature of the sample for these additional control measurements increased quite significantly during the initial phase of the light-scattering measurements, which was also the situation for the measurements on samples exposed to microwaves (Fig. 3d). After some adjustments to better approximate the heating effect of exposure to 20 GHz microwave radiation, we observe that in these cases the $b$-values were also enhanced. Specifically, when an air stream was used to heat the sample above the ambient temperature in a small region surrounding the sampled position, the $b$-value was 2.91 ± 0.11 (Table 2) and yielded $p$ < 10^–4^ for a statistical comparison (Table 3) with the constant ambient temperature control of 34.9 °C. Conversely, $p$-values of 0.89 and 0.20 were recovered when the $b$-values were compared with measurements using 20 GHz and 29 GHz radiation with an input power of 166 mW. Similarly, the studies using an IR laser to generate a tight-temperature gradient at the sample position that mimicked the effect of heating by microwave radiation, yielded a $b$-value was 2.86 ± 0.04 (Table 2) and again $p$ < 10^–8^ in the statistical comparison (Table 3) with the constant ambient temperature control of 34.9 °C. In these cases, $p$-values of 0.09 and 0.003 were recovered when the $b$-values were compared with measurements using 20 GHz and 29 GHz radiation with an input power of 166 mW. The comparison against 29 GHz would have been interpreted as significant with a cut-off of $p$= 0.05, and we suggest this arises from the fact that that the thermal gradient of the IR laser was the steepest in comparison with the 29 GHz thermal gradient (Supplementary Figure S1a). We therefore conclude that enhanced $b$-values, which in the previous section appeared to be a unique characteristic for samples exposed to microwave radiation above 20 GHz in frequency and a power of 166 mW in intensity, can also be recovered from two other experimental configurations in which the sample is heated step-wise. Thus, whereas exposure to microwave radiation in our flow-cell perturbs the kinetics of microtubule growth relative to one-step heating controls in a reproducible manner, this effect cannot be distinguished from the effect of two-step heating controls. Or conversely, although our experimental data cannot conclusively rule-out the possibility of non-thermal effects of microwave radiation on microtubules effecting their growth kinetics, the application of Occam’s razor prescribes that it is not necessary to appeal to anything other than microwave induced heating to explain the experimental data reported here.

## Discussion and conclusions

In this work we sought to establish whether or not the application of microwave fields influences the nucleation and growth kinetics of microtubules over and above well-known effects due to heating. Tubulin is highly polar^35^ and there have been several studies showing that microtubules can align in continuous or oscillating electromagnetic fields^36–39^ including electrophoresis effects^40,41^. Moreover, electric field induced protein structural perturbations have been observed by time-resolved Laue diffraction^42^, albeit at several orders of magnitude higher field-strength than studied here. Conversely, structural studies of whether or not protein structural changes are induced by the application of THz electromagnetic radiation at much lower field-strengths have not always agreed^10,12^.

It is estimated that, on average, there are 26 arrival events and 25 departure events for every net gain of a single tubulin dimer during polymerization^43^. As such, even small perturbations that influence this kinetic balance could potentially perturb the effective forward and backward rate-constants during elongation. For example, given that microtubules align in the presence of oscillating electromagnetic fields^36–41^, at some point entropy perturbations might be expected to influence the polymerization kinetics. Growth assays performed during exposure to microwaves, by their very nature, involve a transient temperature change in the sample^31,32^. Practical considerations led us to focus on the application of microwaves in the low GHz domain, since our device for delivering microwave radiation onto samples within a quartz capillary revealed sample heating within this frequency domain^44^ (Fig. 1b). Although the observation of microwave induced heating gave confidence in the experimental design, the difference in thermal history between the experiment and controls created additional complications. Another limitation of our design is that it took approximately 7 s for samples to transit from ice to the position at which turbidity observations were recorded. It was impractical to shorten this delay by pumping faster or using tubing with a smaller inner diameter, since both these approaches induced bubbles in these viscous samples and bubbles become very problematic for turbidity measurements. Nevertheless, our use of an infrared camera during the application of microwave radiation or other perturbations allowed the temperature at the sample’s position to be monitored with time.

We observed a statistically significant effect of microwave radiation on the growth of microtubules (Fig. 3, Table 3), but this effect is reproduced in experimental geometries which approximate the thermal history associated with the microwave measurements (Figs. 4, 5**, **Table 3). As such, we concur with the conclusion of an earlier study using 2.45 GHz microwave radiation that microwaves do not change the rate of tubulin polymerization into microtubules^20^. The maximum field-strength used in our studies (~ 2 kV/m), while considerably lower than that used in other studies of the influence of electric fields on biomolecules^36,39,42^, is eight orders of magnitude above the signal strength typically associated with mobile telephone networks (~ −60 dBm).

It is possible that our measurements were close to revealing subtle non-thermal effects of microwave radiation yet lacked statistical sensitivity. Under idealized assumptions including that the measured means and standard deviations are true, we can estimate that 200 repeats would be needed to achieve a statistical power of 0.9 for a $p$-value below 0.05 when comparing the effect on $b$-values for 166 mW of 20 GHz radiation against data recorded with an airflow generated thermal gradient across the capillary. However, for reasons of reproducibility, we performed all measurements using protein isolated from a single protein purification, and these preparations are limited to practical volumes given the available equipment. Moreover, not all datasets followed a normal distribution (Supplementary Figure S3), which is a standard assumption underpinning the treatment of experimental errors or sample inconsistencies. Therefore, a completely new design of the instrument utilizing smaller volumes would appear to be required to achieve an order of magnitude higher sensitivity.

There is considerable public interest in the possible existence of non-thermal effects of microwave radiation on biomolecules, due to social concerns about the influence of microwave radiation on human health as appliances operating in this frequency domain become more widely used^1–4^. There is also a highly-speculative literature which argues that microtubules may be able to process information by some unknown mechanism that may relate to consciousness^45–48^. While we consider that these ideas may inspire many to learn more about science, at some point there needs to be a connection between potentially far-reaching concepts and experimental data^49^. In the frequency domain from 3.5 to 29 GHz that we have explored in this work, there is no evidence which points towards any measurable effects on the growth of microtubules that cannot be explained as purely thermally induced.

## Material and methods

### Tubulin sample preparation

Tubulin was extracted and purified from porcine brains, which were provided approximately 40 min after slaughter by Dalsjöfors Meat AB. Brains were transported from the slaughterhouse to the laboratory in ice-cold PBS (Phosphate Buffered Saline) to slow down protein degradation. The protein was purified using two cycles of polymerization-depolymerization in high molarity buffers^50^, with the addition of protease inhibitors (Protease Inhibitor cocktail, 5 ml, Sigma Aldrich) prior to the first centrifugation step. This purification protocol produces tubulin free of microtubule associated proteins (MAPs), which we confirmed using mass spectrometry. Protein solution (General Tubulin Buffer, Cytoskeleton) was divided into aliquots, flash frozen using liquid N_2_, and placed in a −80 °C freezer for storage.

### UV-light scattering measurements

A microspectrophotometer^29^ was used for light absorption measurements of the turbidity of microtubules under the influence of 3.5 to 29 GHz microwave radiation. Data were recorded on an Ocean Optics spectrophotometer. Tubulin polymerization was monitored by measuring the increase in absorbance at 365 nm with time through a 1 mm quartz capillary. Light transmission through the working buffer without tubulin was used as a reference. An increase in absorbance was used as a proxy to indicate the amount of tubulin within microtubules in solution^25,31^. Aliquots of approximately 5 μl tubulin samples were transported from ice to the region of the quartz capillary where the optical density of the sample was measured. The flow was stopped and the 5 μl aliquots were held in the same position for each measurement, and subsequently replaced for the next measurement. The entire system was constructed within an enclosed and insulated Plexiglas box which was temperature controlled by flowing hot air into the chamber. This flow was adjusted to achieve a stable temperature throughout the temperature domain (32 °C to 39 °C) over which light-scattering data were recorded. This temperature domain included the physiological temperature of a living animal, but it could not be too low otherwise the reaction kinetics became too slow to be measured accurately, and could not be too high since otherwise the initial polymerization reaction could occur during the time when the sample was being transferred from ice to the point of measurement. The sample’s temperature was monitored by using a thermocouple near the sample position and using a thermographic camera. The thermographic camera was also used to determine the temperature of the sample during exposure to 3.5 to 29 GHz fields. A delay of 7 s occurred between the sample leaving the ice bath and reaching the position at which the sample’s optical density was measured.

### Exposure to electromagnetic fields

A parallel-plate waveguide-based flow-cell was purpose built to deliver AC fields onto a quartz capillary containing the sample (Fig. 1). The two metal plates were 64 mm long, 5 mm wide and were separated by slightly more than 1 mm, which allowed a quartz capillary sample container to fit between them. One of the plates was made of 0.30 mm thick copper and the other was integrated into the holder. The gap between the plates was supplemented with Rexolite, a polystyrene dielectric suitable for GHz applications, through which a 1 mm diameter hole was drilled to enable a free optical passthrough for the spectrophotometry measurements. A coaxial cable (Stability, Maury Microwave) fed the GHz fields from a signal generator (Anritsu MG3694C) through the waveguide, which was terminated on a 50 Ω resistor. Measurements using a thermographic camera of water heating within a quartz capillary established that there was optimal energy transfer from the AC field generator to the sample at a frequency of 20 GHz. Moreover, the thermographic camera (FLIR A600-Series) was used to identify the region where heating from the 20 GHz fields was maximal (Fig. 1b), where a hole was drilled in the dielectric to allow an optically clear path and the sample’s optical density was recorded at this point. For the experimental runs when an electromagnetic field was applied, the signal generator was turned on for the entire duration of the measurement.

### Measurements of tubulin polymerization

Measurements were carried out both with and without applied microwave fields. Because microwaves induce heating, control measurements were performed with the entire system pre-heated to the desired temperature. Three sets of measurements were made with the applied 20 GHz frequency power being nominally at source, ≤ 10^–5^ mW (no field), nominally 32 mW (15 dBm, 26 mW at waveguide), nominally 80 mW (19 dBm, 66 mW at waveguide) and nominally 200 mW (23 dBm, 166 mW at waveguide), where losses in the apparatus were measured to be 0.8 dBm. The 3.5 GHz and 29 GHz measurements were performed with the highest input power only (nominally 200 mW). A thermographic camera was used to measure the sample’s temperature at the position where the sample’s optical density was measured (Fig. 1b), which was a few degrees warmer than the surroundings when the microwave radiation was applied. The sample reached a stable temperature within seconds of arriving at the measurement spot position. After every measurement the sample was flushed, followed by washing the capillary with Milli-Q water and every new measurement began with a fresh sample.

### Wave guide characterization and modelling

To facilitate comparisons between studies it has been recommended that experimental data is accompanied by computational modelling where appropriate^51^. Full wave electromagnetic simulations were run in Ansys HFSS in order to predict the electric field distribution within the sample. Representative snapshots of a single phase of the simulation shows standing wave patterns of the electromagnetic field inside the capillary (Fig. 1e, Supplementary Figure S2a, b). The sample was modelled as water (with dielectric properties according to the Debye model, specifically a relative permittivity of 78.4 and a dielectric loss tangent of 0.025) at room temperature (300 K), and the coaxial to parallel plate mode converter was not included in the simulation. The field distributions differ between the frequencies in the simulations and thermal line-profiles along the capillary are in keeping with the simulation results since the field intensity varies along the capillary length (Supplementary Figure S1a). A line profile across the capillary shows that the temperature increase is slightly higher in the middle of the capillary, but the projected profile of the capillary cross section has to be taken into account, since the infrared camera will show the capillary walls in projection, which are cooled slightly due to their contact with the surrounding atmosphere (Supplementary Figure S1b). We also measured the transmitted and reflected scattering parameters (S21 and S11) for the RF-device filled with buffer and empty using a Vector Network Analyzer (VNA, Keysight N5247A PNA-X) (Supplementary Figure S2c–d). For these measurements, the SOLT calibration method was used, situating the calibration plane at the device interface with the coaxial cables. The S21 and S11 parameters allow us to calculate the power emission with and without sample of the waveguide and it shows that when buffer is present the emission is considerably higher (Table 1). The reported S values are an average around the specific frequencies (± 0.1 GHz). The difference in emission between the when the capillary was filled with sample and when it was empty suggests a large fraction of the emitted microwave power is absorbed by the sample, especially at the two higher frequencies used in this study. Simulated S21 and S11 parameters are in agreement with the measured values.

We measured the losses in the coaxial cable connecting the signal generator and the waveguide for 20 GHz at the two lower power levels to be 0.8 dBm. Our power meter (Anristu model ML2437A) could not measure above 20 dBm, but we assume that very similar losses apply for across the frequency domain used in this study since the coaxial cable and connector (K-connector) have stable characteristics over the frequency range. We therefore refer to the three different input powers into the waveguide as 26 (14.2 dBm), 66 (18.2 dBm) and 166 mW (22.2 dBm).

The conductivity measurements of the sample buffer used for the SAR estimates were made with a conductivity meter (WVR HCO 304) at room temperature (22 °C) and do not inform us about the frequency dependence of this parameter.

### Control measurements using an infrared laser and airflow heating

Control measurements using other heating mechanisms were performed in a similar fashion as all measurements using microwaves. A fibre-optic cable from an IR laser (1440 nm, LuOcean P2 LU1470C Diode Laser) was positioned inside the measurement chamber such that the IR induced thermal heating profile mimicked that of the microwave heating. By adjusting the power input this could be matched to the absolute temperature change. The IR laser heated spatial profile was somewhat sharper (thermal spatial distribution ~ 3 mm, Supplementary Figure S1a) than that measured for the microwave exposed region when samples enter the device (thermal spatial gradient ~ 7 mm, samples enter from the right in Supplementary Figure S1a). This difference, however, is a small when compared with the length of the sample transfer lines used in this study (~ 10 cm). The essentially flat thermal spatial profiles of 3 GHz and 4 GHz in Supplementary Figure S1a are substituted for measurements of the thermal profile when using 3.5 GHz radiation. Numerous measurements were performed using 3.5 Hz exposure and, like the 3 GHz and 4 GHz measurements, did not show any detectable heating of the sample. However, we did not save thermal spatial profiles from those measurements and these measurements could not be repeated since our thermographic camera could not be restarted when revising this manuscript. Since the polarized IR light could potentially interact with microtubules in an orientation-dependent way, another approach for applying a localized heating was devised. A flow of heated air from a hot air gun (STEINEL HG2320E) was focused and positioned so that the thermal profile (thermal spatial distribution ~ 5 mm, Supplementary Figure S1a) also mimicked that of the microwave heating.

## Supplementary Information

Supplementary Figures.

## Data Availability

The datasets generated during the current study is available from the corresponding author’s GitHub page, https://github.com/Neutze-lab.

## Code availability

The custom code used to process the data and calculating statistics was implemented in MATLAB and is available from the corresponding author’s GitHub page, https://github.com/Neutze-lab.
