# Author Correction: No observable non-thermal effect of microwave radiation on the growth of microtubules

**DOI:** 10.1038/s41598-024-72098-4

**Published:** 2024-09-23

**Authors:** Greger Hammarin, Per Norder, Rajiv Harimoorthy, Guo Chen, Peter Berntsen, Per O. Widlund, Christer Stoij, Helena Rodilla, Jan Swenson, Gisela Brändén, Richard Neutze

**Affiliations:** 1 Department of Chemistry and Molecular Biology, University of Gothenburg, Gothenburg, Sweden; 2 Department of Physics, Chalmers University of Technology, Gothenburg, Sweden; 3 Monash Health Imaging, Monash Health, Clayton, VIC Australia; 4 Institution of Biomedicine, University of Gothenburg, Gothenburg, Sweden; 5 CSTechnologies, Växjö, Sweden; 6 Department of Microtechnology and Nanoscience, Chalmers University of Technology, Gothenburg, Sweden

Correction to: *Scientific Reports* 10.1038/s41598-024-68852-3, published online 07 August 2024

The original version of this Article contained an error in the Author Contributions section,

“RN, GB, GH, PN, RH, GC and JS designed research. C.S. designed and built the device for delivering microwaves. GB, PN and GH isolated and purified active tubulin. GB, PN, GH and GC performed light-scattering measurements. CS and HR contributed analytic tools. GH, PN, RH, GC, POW and RN analysed data. GH, PN, RH and RN wrote the manuscript.”

now reads:

“RN, GB, GH, PN, RH, GC, PB and JS designed research. PB, C.S. designed and C.S built the device for delivering microwaves. GB, PN and GH isolated and purified active tubulin. GB, PN, GH and GC performed light-scattering measurements. CS and HR contributed analytic tools. GH, PN, RH, GC, POW and RN analysed data. GH, PN, RH and RN wrote the manuscript.”

The original Article has been corrected.

