# Supplementary material for: No observable non-thermal effect of microwave radiation on the growth of microtubules
Source: Sci Rep. 2024 Aug 7;14:18286. doi: 10.1038/s41598-024-68852-3 (PMC11306338; doi:10.1038/s41598-024-68852-3)
Supplement: Supplementary file 1 — Supplementary Figures. [file 41598_2024_68852_MOESM1_ESM.pdf]

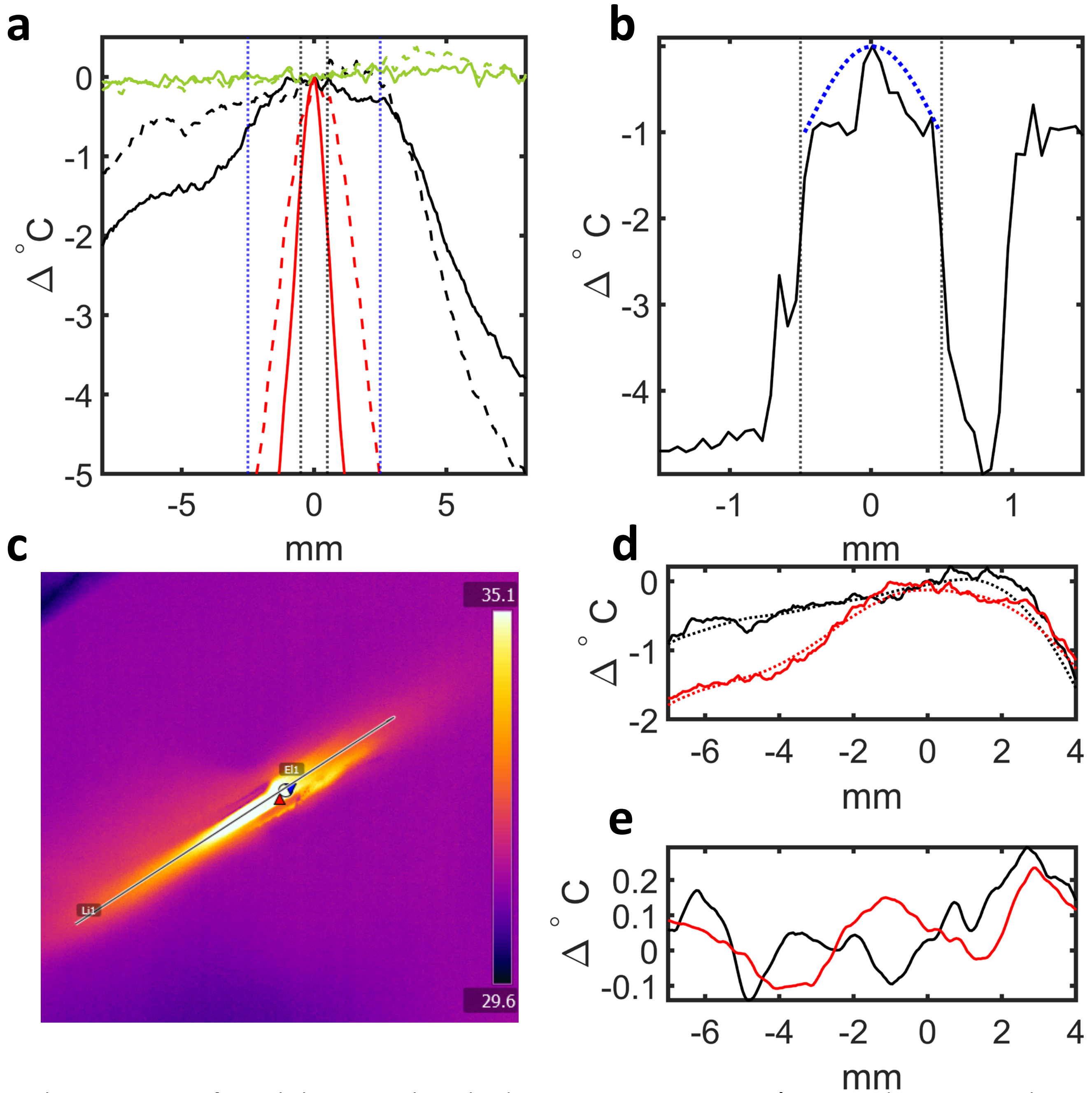

Characterization of sample heating withing the device using an IR camera. **a)** Measured temperature line profiles along the capillary for the 20 GHz (solid black line) and 29 GHz (dashed black line) exposure with input power of 166 mW, as well as the two inhomogeneous heating methods, hot air (dashed red line) and IR laser (solid red line) experimental condition. Thermal line profiles for 3 GHz and 4 GHz are shown in dashed and solid green, since no thermal profiles using 3.5 GHz illumination were saved prior to the thermographic camera failing irrecoverably. Capillary edges are marked with vertical dotted black lines and edge of sample aliquot with vertical dotted blue lines. For these measurements the IR camera was positioned directly in front of the waveguide and the capillary was completely filled with water. Variations in field strength along the capillary are suggested by variations in thermal line profile along the capillary. **b)** Measured temperature gradient across the capillary (solid black line) with an overlay of the shape of the projected cross section of the capillary (dotted blue line). Capillary edges are marked with vertical dotted black lines. The apparent thermal gradient across the capillary is, to some extent, reflecting the curvature of the capillary. **c)** Thermal image from the IR camera when it was positioned at an oblique angle during data collection. Circle marks the area where the mean temperature is recorded during a measurement. **d)** Line profiles of panel a (20 GHz with red solid line and 29 GHz with black solid line). Dotted lines represents the baseline of the line profile extracted using a lowpass filter. **e)** The difference between the solid and dotted lines in panel d show the thermal representation of the standing wave oscillations seen in Figure 1e and Supplementary Figure 2ab.

Supplementary Figure S1: Hammarin *et al.*.

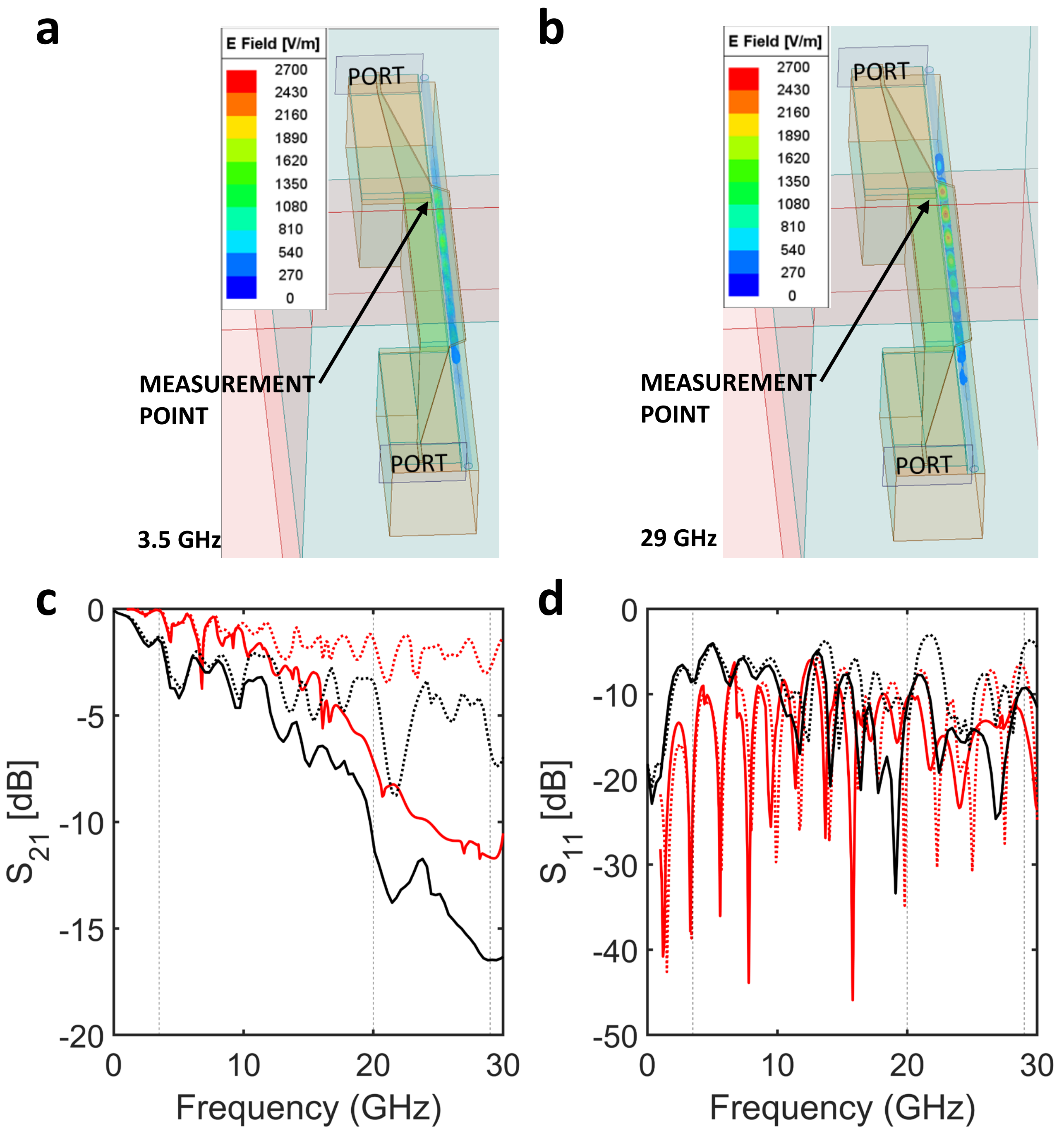

Simulations and measurements of the waveguide**. a-b**) Simulations of electric field inside the capillary for a 1 W input with a frequency of 3.5 GHz (a) and 29 GHz (b) respectively. The sample capillary is shaded light blue. Ports for the simulations are marked. This is a representative snapshot of a single phase during the simulations. The connection between the coaxial and the transmission line is not part of the simulation. **c-d**) Transmitted and reflected scattering parameters (S_{21} (c) and S_{11} (d)) for the RF-device filled with buffer (solid line) and empty (dotted line). Experimental results measured with a Vector Network Analyzer (VNA) shown in black and simulated parameters from the model seen in panel **a-b** are shown in red. Dotted vertical lines indicate the three frequencies of interest, 3.5 GHz, 20 GHz and 29 GHz.

Supplementary Figure S2: Hammarin *et al.*.

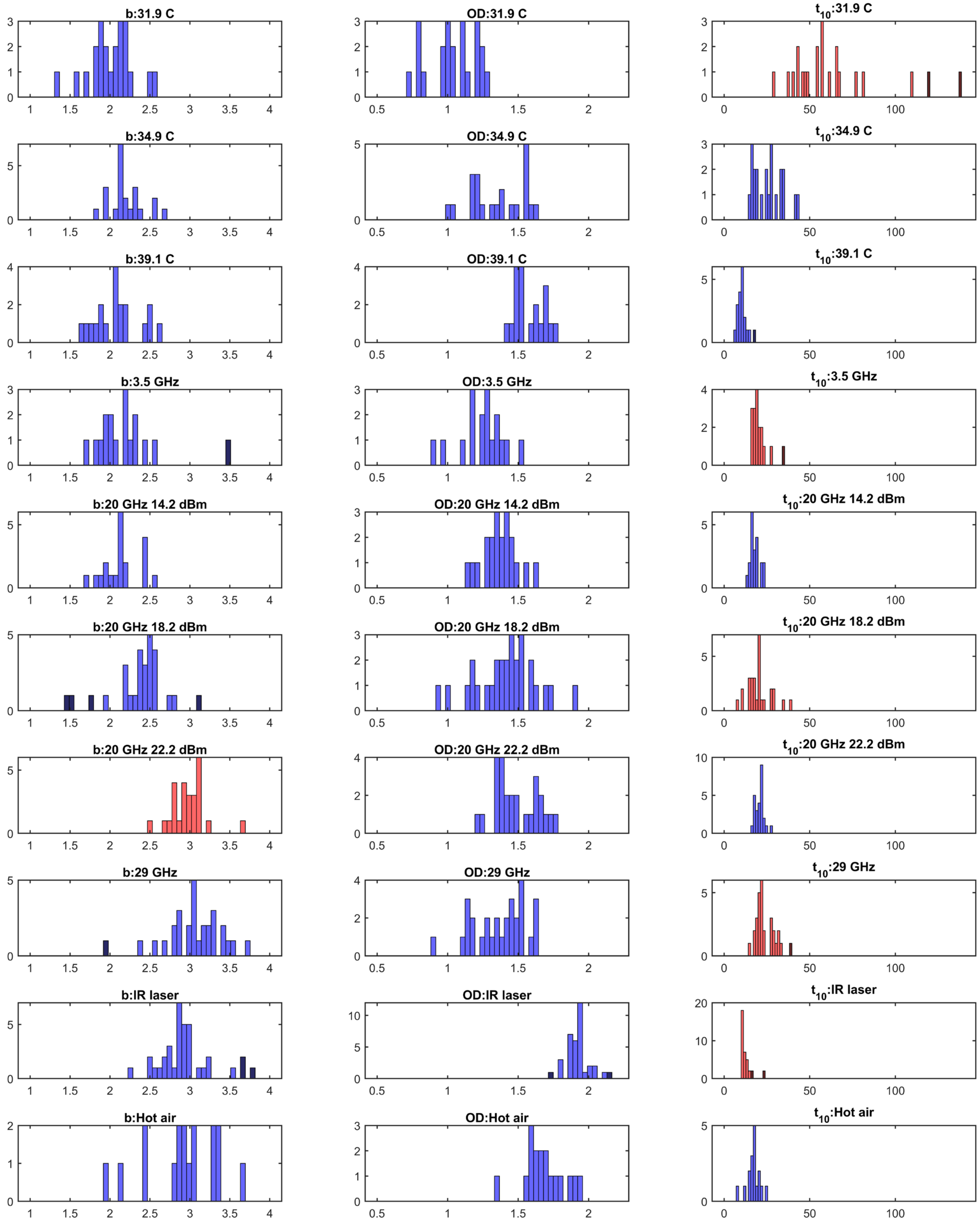

The experimentally characterized distributions for b, OD and t_{10} for the full range of experimental conditions. Outliers identified with the Median Absolute Deviation method (MAD) are marked with black bars in the histograms. Distributions failing one of the applied statistical tests for normality of the parameters (Anderson-Darling (AD), One sample Kolmogorov-Smirnov (KS), Lilliefors (L) and Jarque-Bara (JB)) with a cut-off at $p=0.05$ are marked with red bars. The 31.9 °C t_{10} , 3.5 GHz t_{10} and 20 GHz 22.2 dBm b parameters fails the JB test. The 20 GHz 18.2 dBm t_{10} fails the L test. The 29 GHz t_{10} parameters fails both AD and L test. The IR t_{10} fails all applied tests.

Supplementary Figure S3: Hammarin *et al..*
